# *Pfaffia paniculata* Extract, a Potential Antimicrobial Agent against *Candida* spp., *Pseudomonas aeruginosa*, and *Streptococcus mutans* Biofilms

**DOI:** 10.3390/microorganisms12061165

**Published:** 2024-06-08

**Authors:** Diego Garcia Miranda, Lucas de Paula Ramos, Nina Attik, Thaís Cristine Pereira, Luciane Dias de Oliveira, Maria Cristina Marcucci, Flavia Pires Rodrigues, Graziella Nuernberg Back Brito, Florence Carrouel

**Affiliations:** 1 Multimaterials and Interfaces Laboratory (LMI), CNRS UMR 5615, University Claude Bernard Lyon 1, University of Lyon, 6 rue Victor Grignard, 69622 Villeurbanne, France; nina.attik@univ-lyon1.fr; 2 Faculté d’Odontologie, University Claude Bernard Lyon 1, University of Lyon, 7 rue Guillaume Paradin, 69008 Lyon, France; 3 Laboratory “Health Systemic Process” (P2S), UR4129, Faculty of Medicine Laennec, University Claude Bernard Lyon 1, University of Lyon, 7 rue Guillaume Paradin, 69008 Lyon, France; lucas.de-paula-ramos@univ-lyon1.fr; 4 Department of Biosciences and Oral Diagnosis, Institute of Science and Technology, São Paulo State University, Francisco José Longo 777, São José dos Campos 12245-000, SP, Brazilluciane.oliveira@unesp.br (L.D.d.O.); cristina.marcucci@unesp.br (M.C.M.); 5 Department of Health Sciences, Paulista University, Highway President Dutra km 157, São José dos Campos 12240-420, SP, Brazil; 6 Oral Biology Division, School of Dentistry, Faculty of Medicine and Health, University of Leeds, Leeds LS2 9LU, UK; 7 Christian Life University Foundation, Estrada Municipal do Pinhão do Borba, Pindamonhangaba 12412-825, SP, Brazil; backbritogn@gmail.com

**Keywords:** antifungal agents, antibacterial agents, herbal medicine, *Candida* spp., *Pfaffia paniculata*, Brazilian ginseng

## Abstract

The World Health Organization (WHO) has prioritized developing new drugs against specific bacteria and fungi, such as *Enterobacteriaceae* and *Candida* spp. While *Pfaffia paniculata* is commonly called the “cure-everything”, its scientifically proven benefits are limited to anti-inflammatory and antioxidant actions. Therefore, this study aims to determine the spectrum of antimicrobial activity of *Pfaffia paniculata* and assess its cytotoxicity. Thus, broth microdilution test was conducted according to the CLSI M7-A9 and M27-A3 reference methods. After screening, microbial species with minimum inhibitory concentration (MIC) values were selected for biofilm tests. These tests evaluated biomass using the crystal violet (CV) test, metabolic activity using the MTT assay, and structural analysis via Scanning Electron Microscopy (SEM). Cytotoxicity was evaluated in human gingival fibroblasts (FMM-1). There were reductions of 29.4 and 42.7% in CV and MTT assays for *Candida* spp. biofilm. *S. mutans* and *P. aeruginosa* biofilms showed a decrease of 15.7 and 28.6%, respectively. Cell viability tests indicated 55.1, 56.9, and 65.5% of viability after contact with 1.93, 0.96, and 0.48 mg/mL of the extract, respectively. The *P. paniculata* extract showed antimicrobial action, displayed MIC values, and antibiofilm action on *P. aeruginosa*, *S. mutans,* and *C. albicans*. The cytotoxicity on the FMM-1 cell line was dose-dependent. Therefore, *P. paniculata* extract holds significant potential for developing new drugs.

## 1. Introduction

Antimicrobial resistance (AMR) is one of the top 10 global public health threats [1]. Worldwide, in 2019, AMR is estimated to have caused 1.27 million deaths and contributed to 4.95 million deaths [2]. An increasing number of bacterial, parasitic, and fungal infections can no longer be treated with commonly used drugs [1,2]. For several decades now, the bacteria or fungi responsible for common or serious infections have developed resistance to all the new antimicrobials on the market [3]. *Enterobacteria*, *Staphylococcus*, *Enterococcus,* and fungi of the genus *Candida* are responsible for human infections without pharmacological options for therapy [3,4,5,6,7]. Faced with this situation, the World Health Organization published a list of bacterial species in 2017 [2], followed by a list of fungi in 2022 [8], including *Candida albicans* and *Candida glabrata*, for which the development of new therapeutic options was considered a priority to control morbidity and mortality rates worldwide.

Microorganisms can develop numerous resistance mechanisms or use strategies such as biofilm organization to resist antimicrobial exposure. Biofilms are a structured association of microorganisms such as bacterial and/or fungal cells enclosed in a self-produced extracellular matrix and bonded to a biotic or an abiotic surface. Microorganisms organized within biofilm can use the cell-to-cell communication/signaling scheme known as quorum sensing [9,10] and, thus, be more “recalcitrant” to antimicrobial agents [11]. These biofilm characteristics lead to an exponential increase in the resistance of microorganisms, making the dispersion of drugs much more difficult and increasing resistance by up to 1000 times compared to planktonic cells [11,12,13,14,15] in AMR and the failure of conventional therapies. Therefore, new molecules are needed to combat these pathogens [16]. Consequently, the use of phytotherapy in the treatment of diseases has gained prominence due to its essential biological effects [17,18,19]. Natural products can regulate key pathophysiological processes linked to human diseases, such as inflammation, hypoxia, fibrosis, angiogenesis, oxidative stress, cell proliferation, migration, and metabolism [20]. They also present antibacterial and anti-biofilm actions [21,22,23,24,25]. *Pfaffia paniculata*, popularly known as Brazilian ginseng, is a plant native to the Neotropical region [26,27] considered as a source of antimicrobial compounds with antioxidant, analgesic, and anti-inflammatory properties [28] and registered in the Natural Products Alert database for plants used against cancer [29,30]. This root belongs to the *Amaranthaceae* family and is composed of several components: saponins (named pfaffosides A, B, C, D, E, and F), pfaffic acid, stigmasterol, sitosterol, and glycosides allantoin, β-ecdysterone, stigmasterol, and flavonoids [27,28,31,32,33,34,35,36]. The six classes of saponins exhibit demonstrated antitumor and anti-inflammatory effects, while other components promote cell proliferation, tissue regeneration, and skin protection. Additionally, they enhance hydration and provide anti-inflammatory benefits [37,38,39].

Thus, *P. paniculata* could be an efficient antimicrobial alternative to conventional antibiotics and antifungal treatments. This study aims to answer the following research question: does *P. paniculata* exhibit antifungal and antibacterial activity against *Candida* and bacterial species and is it nontoxic to human fibroblasts (FMM-1)? The objectives were to investigate the antibiofilm activity of the glycolic extract of *P. paniculata* against six *Candida* species (*C. albicans*, *C. dubliniensis*, *C. glabrata*, *C. guilhermondii*, *C. krusei*, and *C. tropicalis*) and five bacterial species (*E. faecalis*, *P. aeruginosa*, *S. aureus*, *S. epidermidis*, and *S. mutans*) and to evaluate its cytotoxic effects on human fibroblasts (FMM-1).

## 2. Materials and Methods

### 2.1. The Chemical Reagents

Glycolic extract of *P. paniculata* (lot: PRODO19544, Mapric Greentech Company^®^, São Paulo, Brazil); ethanol (CAS nº: 64-17-5, purity: 99.5%, Synth^®^, Diadema, Brazil); Mueller Hinton broth (Becton Dickinson^®^, Franklin Lakes, NJ, USA); brain heart infusion agar (Becton Dickinson^®^, Franklin Lakes, NJ, USA); Sabouraud infusion agar (Becton Dickinson^®^, Franklin Lakes, NJ, USA); sterile saline solution (0.9% NaCl) (LGC Biotechnology^®^, Cotia, Brazil); Roswell Park Memorial Institute—RPMI medium (with glutamine, without bicarbonate, phenol red indicator, and MOPS pH 7.0 ± 0.1 (Becton Dickinson^®^, Franklin Lakes, NJ, USA)); violet crystal (CAS nº 548-62-9 purity: 97.5%, Sigma-Aldrich^®^, St. Louis, MO, USA); acetic acid (CAS nº 64-19-7 purity: 97.5%, Sigma-Aldrich^®^, St. Louis, MO, USA); 3-(4,5-Dimethyl-2-thiazolyl)-2,5-diphenyl-2H-tetrazolium bromide powder (MTT) (CAS nº: 298-93-1, purity: 97.5%, Sigma-Aldrich^®^, St. Louis, MO, USA); dimethyl sulfoxide (DMSO) (CAS nº: 67-68-5, purity: 99.9%, Sigma-Aldrich^®^, St. Louis, MO, USA); Eagle’s medium modified by Dulbecco (DMEM) (LGC Biotechnology^®^, Hoddesdon, UK); Fetal Bovine Serum (FBS) (Invitrogen^®^, New York, NY, USA); and neutral red (CAS nº 553-24-2 purity: 90%, Sigma-Aldrich^®^, St. Louis, MO, USA).

### 2.2. Equipment

Analytical balance (Mettler Toledo^®^, Balance XPR106DUH/A, Columbus, OH, USA); drying and sterilization oven (CQA Química Americana LTDA^®^, Paulinia, São Paulo, Brazil); stirrer (Miulab^®^, Micro plate shaker MIX-1500, Hangzhou, China); water bath precision (Termo Fisher Scientific^®^ TSGP02, Waltham, MA, USA); spectrophotometer (Lonza Biotek^®^, ELX808LBS, Winooski, VT, USA); and CO_2_ incubator (Sanyo^®^, MCO-19AIC(UV)^®^, Osaka, Japan).

### 2.3. Plant Extract

The glycolic extract of *P. paniculata* was obtained from Mapric Greentech Company® (São Paulo, Brazil), with the appropriate certification reports from the manufacturer. According to the company, the extract is produced by mixing the root powder in 70% alcohol. After 24 h of extraction, the extract was lyophilized and diluted in propylene glycol. The concentration was confirmed through soluble solids content analysis, indicating a concentration of 1.93% (1.93 mg/mL).

### 2.4. Microbial Strains

Reference strains of American Type Culture Collection (ATCC) were used to test the antimicrobial activity: *Candida albicans* (ATCC 18804), *Candida dubliniensis* (ATCC MYA 646), *Candida glabrata* (ATCC 9030), *Candida guilhermondii* (ATCC 6260), *Candida krusei* (ATCC 6258), *Candida tropicalis* (ATCC 13803), *Enterococcus faecalis* (ATCC 4083), *Pseudomonas aeruginosa* (ATCC 15442), *Staphylococcus aureus* (ATCC 6538), *Staphylococcus epidermidis* (ATCC 12228), and *Streptococcus mutans* (ATCC 35688).

### 2.5. Antifungal and Antibacterial Action of P. paniculata against Planktonic Microorganisms

The referenced methods CLSI M07-A10 for dilution antimicrobial susceptibility tests for bacteria [40] and CLSI M27-A3 for broth dilution antifungal susceptibility testing of yeasts [41] were used to determine the minimum inhibitory concentration (MIC) and minimum microbicidal concentration (MMC) of *P. paniculata* extracts.

Strains were cultured in brain heart infusion (bacteria) or Sabouraud (Yeast) agar at 37 °C, pH 6.8–7.0, with 5% of CO_2_ (for *S. mutans*), for 24 h. For each strain, bacterial inoculums were prepared in sterilized 0.9% NaCl solution and standardized at 10^6^ colony-forming units (CFU)/mL.

In parallel, serial dilutions of the *P. paniculata* extracts (initial concentrations: 1.93, 0.96, and 0.48 mg/mL) were prepared in a plate. Each of 10 serial dilutions was a 1:2 dilution of the prior concentration in 100 µL of Mueller Hinton broth for bacteria and RPMI 1640 medium.

Then, 100 µL of standardized inoculum was added to each well. After incubation for 24 h at 37 °C and 5% of CO_2_ (for *S. mutans*), the MIC value was determined. It corresponded to the concentration of the first well without microbial turbidity, next to the well showing apparent microbial growth.

At a second time, to determine MMC values, a 10 µL aliquot of each well was inoculated into BHI or Sabouraud agar. After 48 h of incubation at 37 °C and 5% of CO_2_ (for *S. mutans*), the MMC was determined. It corresponded to the concentration of the well where there was no microbial growth and where the concentration of *P. paniculata* extract was the lowest.

### 2.6. Antifungal and Antibacterial Action of P. paniculata against Monotypic Biofilms

Only species for which an MIC was determined were tested. Monotypic biofilms were prepared in 96-well microplates using standard suspensions containing 10^7^ CFU/mL incubated at 37 °C, 5% CO_2_ (for *S. mutans*), and matured for 48 h, with replacement of the medium for each 24 h.

Three concentrations of *P. paniculata* extract were tested: 1.93 mg/mL, 0.96 mg/mL, and 0.48 mg/mL. The positive control for bacteria was a solution of chlorhexidine digluconate at 0.12%, while the positive control for yeast was a solution of nystatin at 100,000 units/mL. The negative control consisted of a sterile saline solution.

Monotypic biofilms were exposed to each solution for 5 min, and the final volume in each well was 200 µL. Subsequently, solutions were discarded and biofilm was washed twice with sterile saline solution to remove dead cells. Then, the biofilms were analyzed to evaluate biomass (by crystal violet staining) or metabolic activity (by MTT test).

### 2.7. Evaluation of the Structure of the Monotypic Biofilms (Biomass) Using Crystal Violet Staining

Monotypic biofilms were fixed with 200 µL of methanol per 20 min. Then, the solution was removed and the plate was incubated at 37°C for 24 h for drying. Crystal violet (CV) (1% *v*/*v*) was added for 5 min; then, the dye was removed and the wells were washed with sterile saline solution and 33% acetic acid. The plate was read in a spectrophotometer at a wavelength of 570 nm, and optical density (OD) values were converted to biofilm biomass, considering OD of the treated group multiplied by 100 divided by the mean OD of the control group.

### 2.8. Evaluation of the Metabolic Activity of Monotypic Biofilms by MTT Assay

MTT solution was applied to monotypic biofilms during 1 h at 37 °C. After incubation, the solution was removed and 200 µL of DMSO was added into the wells and the plates were incubated again at 37 °C for 10 min and placed on the stirrer, under constant agitation for 10 min. The OD were read using a spectrophotometer at a wavelength of 570 nm, and OD values were converted to the metabolic activity of the microorganisms on monotypic biofilms, considering OD of the treated group multiplied by 100 divided by the mean OD of the control group.

### 2.9. Scanning Electron Microscopy (SEM)

For SEM, the monotype biofilms of each species were cultured on acrylic discs. These discs were arranged in 24-well plates with standardized suspensions at 10^7^ CFU/mL of microorganism and the BHI culture medium. After 48 h of growth at 37 °C, the biofilms were treated with 1.93 mg/mL of *P. paniculata* extract or 0.9% NaCl for 5 min. After, biofilms formed on acrylic discs were subjected to fixation with 2.5% glutaraldehyde for 1 h and then submerged in 6 alcohol concentrations (10, 25, 50, 75, 90, and 100%) for 20 min. The plates were incubated at 37 °C for 24 h to dry the discs before being transferred to aluminum stubs and covered with gold for 120 sec at 40 mA. After the drying process, the discs were analyzed and photographed by SEM.

### 2.10. Evaluation of Cytocompatibility with P. paniculata Extract

#### 2.10.1. Preparation of Sample Plates

Human gingival fibroblasts (FMM-1) were cultured in DMEM supplemented with 10% FBS, incubated at 37 °C and 5% of CO_2_. Cytotoxicity assay was performed with 4 × 10^4^ viable cells, plated on a 96-well microplate, and incubated at 37 °C and 5% CO_2_ for 24 h. After cell adhesion, the supernatant was removed to apply *P. paniculata* extract in concentrations of 0.48, 0.96, and 1.93 mg/mL for 5 min. The control group received only DMEM + 10% FBS treatment for the same period. Each experimental group was carried out with n = 10 and two independent repetitions. After, wells were washed twice with sterile saline solution. Then, the plates were used for cell viability tests (metabolic activity, membrane integrity, and lysosomal activity).

#### 2.10.2. Evaluation of Metabolic Activity of Fibroblasts (FMM-1) by MTT Assay

MTT solution was prepared by adding 0.5 mg of MTT powder to 1 mL of sterile PBS. A hundred microliters of MTT solution was put in each well of the sample plates, which were incubated (37 °C, 5% CO_2_) for 1 h, protected from light. The solution was discarded and 100 µL of DMSO added to each well to expose the formazan crystals metabolized by viable cells. After 10 min of incubation and 10 min of agitation in a stirrer, the absorbance of the wells was read on a spectrophotometer under a wavelength of 570 nm.

#### 2.10.3. Evaluation of Membrane Integrity Using the Crystal Violet Assay on Fibroblasts (FMM-1)

CV solution was prepared with 0.2 mg of dye powder per milliliter of PBS. A total of 100 µL of 10% formaldehyde was added to each well of the sample plates, and the cells were fixed for 10 min. Next, CV suspension 0.2% was distributed at 100 µL/well for 15 min of incubation, protected from the light. After this period, the wells were washed with distilled water until there was no dye. Subsequently, 100 µL/well of ethyl alcohol at 100% was added, and the microplate was taken to the stirrer for 10 min and read in a spectrophotometer under a wavelength of 570 nm. The OD obtained were converted to a percentage of cell viability since the ability of the dye to bind negative charges is directly proportional to the presence of living cells.

#### 2.10.4. Evaluation of Lysosomal Activity by Neutral Red Assay on Fibroblasts (FMM-1)

This assay aims to assess the incorporation of neutral red into lysosomes. For this, a neutral red solution was prepared with 20 μg of neutral red dye powder per milliliter of PBS. A total of 100 µL of neutral red solution was added to each well of the sample plates, and the plate was incubated for 2 h at 37 °C with 5% CO_2_ and protected from light. The plates were washed twice with distilled water and 100 µL/well of ethyl alcohol at 100% was added. The plate was taken to the stirrer for 15 min and read in a spectrophotometer under a wavelength of 570 nm. OD were converted to a percentage of cell viability, considering OD of the treated group multiplied by 100 divided by the mean OD of the control group.

### 2.11. Statistical Analysis

The data obtained were analyzed for normality using the D’Agostino, Shapiro–Wilk and Kolmogorov–Smirnov tests. Data with normal distribution were analyzed using the one-way ANOVA method complemented by Tukey test. Data without normal distribution were analyzed using the Kruskal Wallis test supplemented by Dunn’s. Significance levels were *p* < 0.0332 (*), *p* < 0.0021 (**), *p* < 0.0002 (***), and *p* < 0.0001 (****). Statistical analysis was carried out using GraphPad Prism 9.0 software.

## 3. Results

### 3.1. Antimicrobial Inhibitory and Bactericidal Activity of P. paniculata Extracts against Planktonic Microorganisms

*P. paniculata* extract obtained MIC for all tested microorganisms, as shown in Table 1. *E. faecalis* and *S. epidermidis* showed growth inhibition with 0.12 mg/mL of *P. paniculata* extract. *C. albicans*, *C. dubliniensis*, *C. glabrata*, *C. guilhermondii*, *C. tropicalis*, *P. aeruginosa,* and *S. mutans* showed growth inhibition with 0.24 mg/mL of *P. paniculata* extract. *C. krusei* and *S. aureus* showed inhibition with 0.48 mg/mL of *P. paniculata* extract.

MMC found for *C. tropicalis* and *P. aeruginosa* was 0.24 mg/mL. For *C. albicans*, *C. dubliniensis*, *C. glabrata*, *C. guilhermondii*, *C. krusei,* and *S. mutans*, it was 0.48 mg/mL. Although, MMC was not found for *E. faecalis*, *S. aureus*, and *S. epidermidis*.

### 3.2. Biomass Density of Monotypic Biofilm after Treatments with P. paniculata Extracts

**Figure 1:**
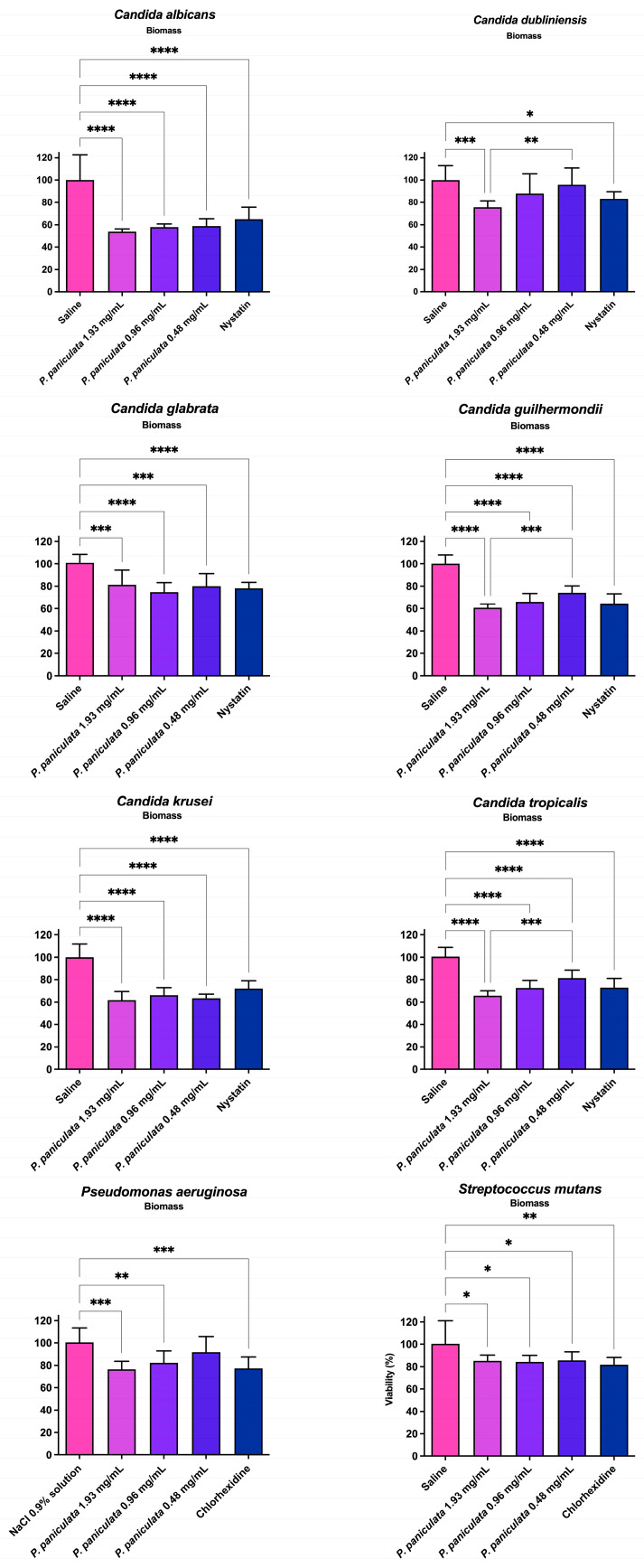
Biomass reduction of *Streptococcus mutans*, *Pseudomonas aeruginosa*, *Candida albicans* and *Candida dubliniensis*, *Candida glabrata*, *Candida guilhermondii*, *Candida krusei,* and *Candida tropicalis* promoted by *Pfaffia paniculata* extracts. Glycolic extract of *Pfaffia paniculata* at concentrations of 1.93, 0.96, and 0.48 mg/mL; *Chexidine digluconate* 0.12% (antifungal positive control); nystatin (antibacterial positive control). * *p* < 0.0332, ** *p* < 0.0021, *** *p* < 0.0002, and **** *p* < 0.0001.

Table 2 and Figure 1 illustrate the reduction rates in exopolysaccharide structural density (biomass) following a 5 min treatment with concentrations of 1.93, 0.96, and 0.48 mg/mL of *P. paniculata* extract, alongside nystatin (100,000 units/mL, positive control for *Candida* species) and chlorhexidine (0.12%, positive control for bacteria).

The reduction in biomass observed after treatment with *P. paniculata* extract at 1.93 mg/mL was greater than after treatment with nystatin (positive control) for *Candida* species.

Similarly, the reduction in biomass observed after treatment with *P. paniculata* extract at 1.93 mg/mL was greater than that observed after treatment with chlorhexidine 0.12% (positive control) for *P. aeruginosa* but lower for *S. mutans*.

### 3.3. Metabolic Activity Reductions of Monotypic Biofilms after Treatments with P. paniculata Extracts

**Figure 2:**
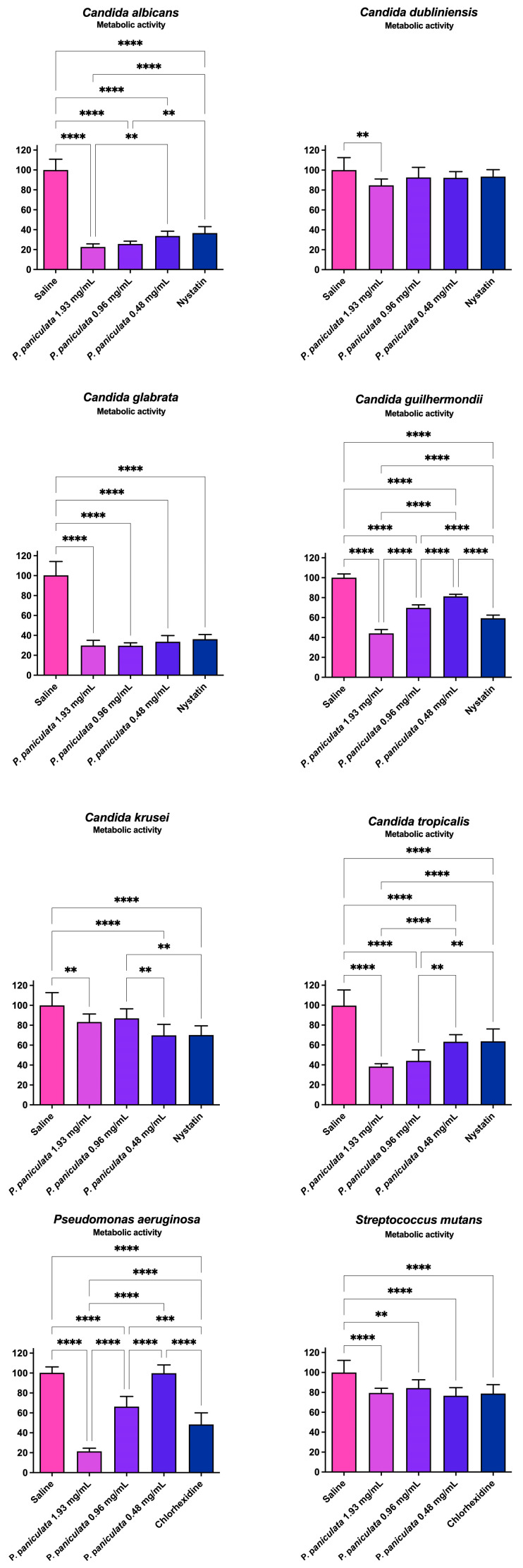
Reduction in metabolic activity of *Pseudomonas aeruginosa, Streptococcus mutans, Candida albicans,* and *Candida dubliniensis* biofilm promoted by *Pfaffia paniculata* extract. Glycolic extract *Pfaffia paniculata* at concentrations of 1.93, 0.96, and 0.48 mg/mL; (antifungal positive control); nystatin (antibacterial positive control). ** *p* < 0.0021, *** *p* < 0.0002, and **** *p* < 0.0001.

Table 3 and Figure 2 display the percentage reduction in metabolic activity of monotypic biofilms following a 5 min treatment with concentrations of 1.93, 0.96, and 0.48 mg/mL of *P. paniculata* extract, as well as nystatin (100,000 units/mL, positive control for Candida species) or chlorhexidine (0.12%, positive control for bacteria).

The metabolic activity reduction observed after treatment with *P. paniculata* extract at 1.93 mg/mL was greater than that observed after treatment with nystatin (positive control) for all the *Candida* species tested except *C. krusei*.

Similarly, the metabolic activity reduction observed after treatment with *P. paniculata* extract at 1.93 mg/mL was greater than that observed after treatment with chlorhexidine 0.12% (positive control) for *P. aeruginosa* but lower for *S. mutans*.

### 3.4. Observation of the Modifications of Monotype Biofilms by Scanning Electron Microscopy

**Figure 3:**
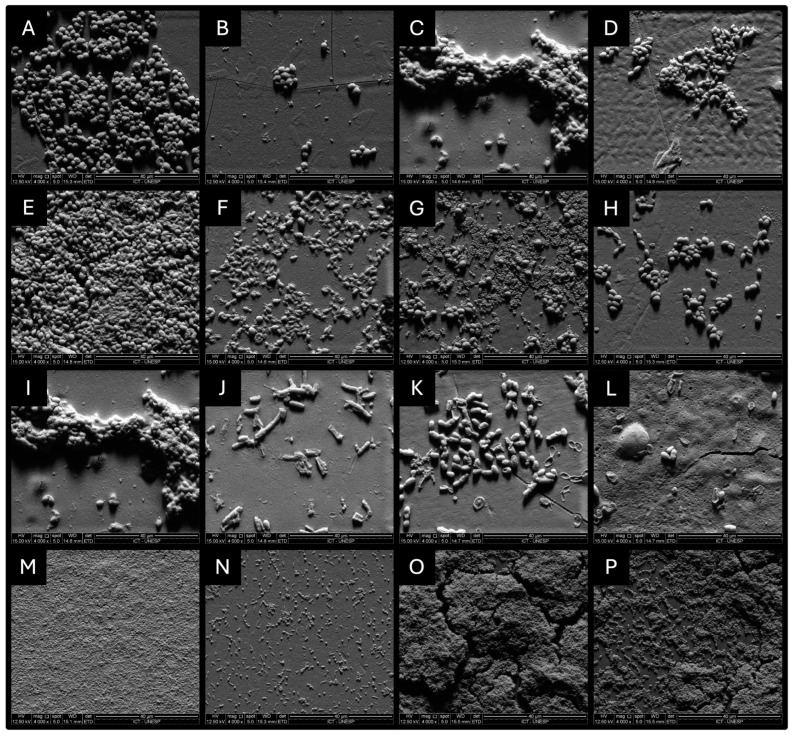
Biofilms of *C. albicans*, *C. dubliniensis*, *C. glabrata*, *C. guilhermondii*, *C. krusei*, *C. tropicalis*, *P. aeruginosa,* and *S. mutans* subjected to analysis by SEM. (**A**) Control group of *C. albicans*, (**B**) *C. albicans* exposed to 1.93 mg/mL *P. paniculata* extract during 5 min, (**C**) control group of *C. dubliniensis*, (**D**) *C. dubliniensis* exposed to 1.93 mg/mL *P. paniculata* extract during 5 min, (**E**) control group of *C. glabrata*, (**F**) *C. glabrata* exposed to 1.93 mg/mL *P. paniculata* extract during 5 min, (**G**) control group of *C. guilhermondii*, (**H**) *C. guilhermondii* exposed to 1.93 mg/mL *P. paniculata* extract during 5 min, (**I**) control group of *C. krusei*, (**J**) *C. krusei* exposed to 1.93 mg/mL *P. paniculata* extract during 5 min, (**K**) control group of *C. tropicalis*, (**L**) *C. tropicalis* exposed to 1.93 mg/mL *P. paniculata* extract during 5 min, (**M**) control group of *P. aeruginosa*, (**N**) *P. aeruginosa* exposed to 1.93 mg/mL *P. paniculata* extract during 5 min, (**O**) control group of *S. mutans*, and (**P**) *S. mutans* exposed to 1.93 mg/mL *P. paniculata* extract during 5 min.

The images obtained indicated morphological alterations and/or a decrease in microbial cells, suggesting that all microorganisms suffered damage after the application of *P. paniculata* extract. The group of *C. albicans* exhibited a significant decrease in amount. Furthermore, it is noteworthy that the surface of the yeast appeared rough and dry after 1.93 mg/mL of *P. paniculata* extract (Figure 3B).

The groups of *C. glabrata* (3F), *C. guilhermondii* (3H), *C. krusei* (3J), and *C. tropicalis* (3L) that received the application of 1.93 mg/mL of *P. paniculata* extract indicate a significant decrease in the number of yeast cells.

The groups of *S. mutans*, *P. aeruginosa,* and *C. dubliniensis* showed visible reductions in the number of cells comprising the biofilm (Figure 3).

### 3.5. Gingival Fibroblast Cell Viability Assayed by MTT, Crystal Violet, and Neutral Red

**Figure 4:**
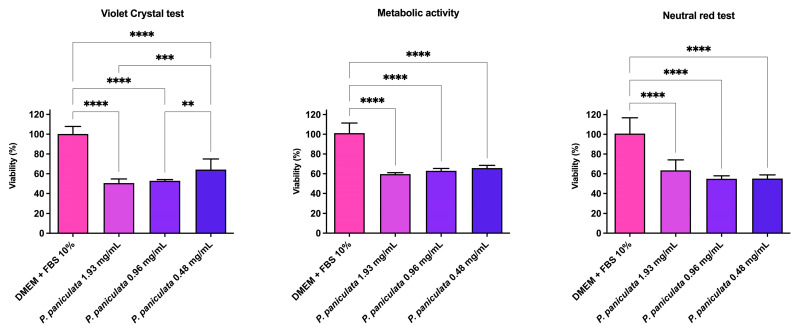
Cytotoxic effects of *P. paniculata* extract on FMM-1 lineage, evaluated for crystal violet, MTT tests, and neutral red. Glycolic extract *P. paniculata* at 1.93, 0.96, and 0.48 mg/mL concentrations. ** *p* < 0.0021, *** *p* < 0.0002, and **** *p* < 0.0001.

The results of cell viability are presented in Figure 4. CV assay indicated that the viability of FMM-1 was of 50.6%, 52.7%, and 64.2% after exposure to 1.93, 0.96, and 0.48 mg/mL of *P. paniculata* extract. MTT assay revealed that the viability of FMM-1 was of 59.6, 63.1, and 65.8% after exposure to 1.93, 0.96, and 0.48 mg/mL of *P. paniculata* extract. Similarly, the neutral red assay showed that the viability of FMM-1 was of 63.5%, 54.9%, and 55.2% after exposure to 1.93, 0.96, and 0.48 mg/mL of *P. paniculata* extract.

## 4. Discussion

The results obtained in this study demonstrated significant antimicrobial potential of *P. paniculata*, mainly regarding its antifungal effects. Additionally, only low cytotoxicity was observed in contact with gingival fibroblasts, with no significant structural damage. Taken together, these results provide new insight into the importance of *P. paniculata* as a therapeutic agent and suggest that *P. paniculata* could represent an important anti-biofilm agent to counteract antimicrobial resistance.

Firstly, the analysis of control groups reveals that some species of *Candida* exhibited higher biomass activity, while others displayed higher metabolic activity. Specifically, *C. krusei* and *C. dubliniensis* biofilms showed higher biomass production. However, these results differ from those reported by Marcos-Zambrano et al. [42], where the comparison between XTT (2,3-Bis-(2-Methoxy-4-Nitro-5-Sulfophenyl)-2H-Tetrazolium-5-Carboxanilide) and CV assays indicated that *C. tropicalis* and *C. albicans* produced more biomass. For *C. glabrata*, the results of both studies were similar and showed that the species have a higher metabolic activity than biofilm density.

Secondly, since it is often difficult to identify the causative agent at the time of clinical diagnosis, broad-spectrum antimicrobial agents are needed for effective treatment. In particular, the broad spectrum of *P. paniculata* could be of great interest in the treatment of candidiasis, for which identification of the fungus is very difficult. In previous studies, *P. paniculata* demonstrated its antimicrobial action on *Klebsiella pneumoniae* [43], *S. aureus, S. epidermidis*, and *Cutibacterium acnes* biofilms [44]. In the present study, *P. paniculata* demonstrated an antibacterial action against *P. aeruginosa* and *S. mutans* and an antifungal action against monotypic biofilms of *C. albicans*, *C. dubliniensis*, *C. glabrata*, *C. guilhermondii*, *C. krusei,* and *C. tropicalis.* These results are in agreement with those of Costa et al. [45] and Ferreira et al. [46] that pointed out that the biomass of *C. glabrata* biofilm presented a lower density compared to other species of *Candida* due to the composition of the biofilm of the species, which counts only with fungal cells and polymer matrix, without the presence of hyphae or pseudo-hyphae. However, *C. glabrata* must have other evasion mechanisms, considering that the structure of its less complex biofilm did not demonstrate greater sensitivity to the antifungal effect of *Pfaffia* extract when compared to *C. dubliniensis* and *C. albicans*.

Thirdly, regarding the action of *P. paniculata* extract on biofilm-organized microorganisms, *C. albicans*, *C. glabrata*, *C. tropicalis,* and *C. guilhermondii* showed greater reductions in metabolic activity than the other monotypic biofilms tested, while *C. krusei* showed a greater reduction in biomass. *C. dubliniensis* showed a variable result in comparing the tests (CV × MTT) according to the concentration of the extract. The concentrations of 1.93 and 0.96 mg/mL showed greater action on the biomass, while the concentration of 0.48 mg/mL showed greater action on the enzymatic activity, with a greater reduction in cellular metabolism. The MTT assay results indicated that the *P. paniculata* extract showed antibiofilm action against *P. aeruginosa*, *S. mutans,* and *Candida* spp. The most significant reductions observed in biofilm viability were 78.6% against *P. aeruginosa*, followed by 77.4% for *C. albicans*.

One possible hypothesis for the antifungal action of *P. paniculata* is related to its main constituents, the saponins called pfafosides. Pfafosides are glycosylated triterpenes, typically composed of five sugar molecules that can interact with sterols present in the fungal cell membrane. This saponin–sterol interaction promotes rearrangements in the fungal cell structure modifying its permeability [47,48,49,50,51]. Additionally, monoterpenes can act on the lipid bilayer, altering its fluidity and transport of substances across the membrane [48].

Comparing the data indicated by Morrissey and Osbourn [47] and Pham et al. [48] regarding saponins with the antimicrobial results obtained by this study, it appears that pfafoside molecules have a greater effect on fungal cells than bacterial ones.

Fourthly, regarding the cytotoxicity of *P. paniculata*, there are few studies, particularly in terms of assessing cellular structure, metabolic activity, or determining the lethal dose. Most research focuses on its anti-inflammatory effects, as it is commonly used for this purpose in traditional medicine [31,49]. A Cochrane review concluded that *P paniculata* had few cytological effects but with a low or very low level of evidence [52]. On the other hand, the current study evaluated the effects of the *P. paniculata* glycolic extract in FMM-1 cells, demonstrating that concentrations of 1.93, 0.96, and 0.48 mg/mL reduced fibroblast cell viability from 34.2% to 49.4%. Cell viability following treatment with *P. paniculata* was similar in all three colorimetric assays used. The metabolic activity (MTT) of the fibroblasts was reduced after contact with concentrations of 1.93, 0.96, and 0.48 mg/mL of the extract. This reduction was also noted in the crystal violet test, which assesses the integrity of the cell membrane and DNA. When assessing lysosomal activity using the neutral red test, it is possible to see that the extract has a protective action on the organelle, where it is possible to see an increase in viability as the concentration of *P. paniculata* increases. These data lead us to believe that the extract promotes a decrease in the number of cells in vitro, reflected in the lower pigmentation of the membrane structure (CV) as well as the decrease in metabolic activity but, when the organelle structure, such as lysosomes, is analyzed, it remains preserved. Although in vitro results showed that the components of the extract decreased cell viability, it is important to notice that these *P. paniculata* concentrations used to evaluate the cytotoxicity were at least twice the MIC values observed for the various microorganisms tested, except for *C. krusei* and *S. aureus*, for which the MIC was 0.48 mg/mL. In addition, at the tested concentrations, *P. paniculata* application is widely recognized both in the scientific community and in traditional medicine [31,49,53].

Eberlin et al. [53] used a dermo-cosmetic, based on *P. paniculata* and two other plants (*Ptychopetalum olacoides* B. and *Lilium candidum* L.), in 21 patients to evaluate the ability of the skin to reduce dark patches around the eyes. The cosmetic was effective in 90% of the cases and did not cause any adverse reactions in any patient, providing a different perspective on toxicity in the context of this study. It must be highlighted that the cells used in both studies were different, and different results could be expected.

Finally, the correlation between cytotoxicity data and antimicrobial activity demonstrates promising applicability of the extract. *P. paniculata* is a broad-spectrum antimicrobial that acts against bacteria (*S. mutans*, *P. aeruginosa*, *Klebsiella pneumoniae* [43], *S. aureus* [44], *S. epidermidis* [44], and *Cutibacterium acnes* [44]) as well as several fungi (*C. albicans*, *C. glabrata*, *C. dubliniensis*, *C. krusei*, *C. tropicalis*, and *C. guilliermondii*). It can act against both planktonic and biofilm microorganisms, which is very interesting since microorganisms organized in a biofilm are more resistant [54]. In addition, the low toxicity of *P. paniculata* confirms that it represents a very good candidate in the fight against resistant microorganisms. Consequently, *P. paniculata* could represent a new therapeutic option to control morbidity and mortality worldwide due to bacteria and fungi listed by the WHO, including *C. albicans* and *C. glabrata* [8].

## 5. Conclusions

The extract of *P. paniculata* exhibited superior antimicrobial efficacy compared to conventional pharmaceuticals, at the doses used in this study (1.93, 0.96, and 0.48 mg/mL), which are very high compared to commonly used antimicrobials doses. It acted against a range of pathogens, including *S. mutans*, *P. aeruginosa*, *C. albicans*, *C. glabrata*, *C. dubliniensis*, *C. krusei*, *C. tropicalis*, and *C. guilliermondii*. Importantly, while the cytotoxicity of the extract was concentration-dependent, human gingival fibroblast viability remained above 55%. These findings suggest that *P. paniculata* extract could serve as a potent alternative to conventional antimicrobials, offering a potential solution to the growing issue of antimicrobial resistance. Nonetheless, further investigation is required to validate these in vitro findings with in vivo studies. Additionally, elucidating the phytochemical components and their mechanisms of action is crucial. These steps are necessary to advance the development of novel therapeutic agents.

## Figures and Tables

**Table 1:** Minimum inhibitory concentration (MIC) and minimum microbicidal concentration (MMC) of *Pfaffia paniculata* glycolic extract for microorganism species. Absent: there were no minimum microbicidal concentrations among the tested concentrations.

*Pfaffia paniculata* Glycolic Extract		
Microorganisms	MIC (mg/mL)	MMC (mg/mL)
*C. albicans*	0.24	0.48
*C. dubliniensis*	0.24	0.48
*C. glabrata*	0.24	0.48
*C. guilhermondii*	0.24	0.48
*C. krusei*	0.48	0.48
*C. tropicalis*	0.24	0.24
*E. faecalis*	0.12	Absent
*P. aeruginosa*	0.24	0.24
*S. aureus*	0.48	Absent
*S. epidermidis*	0.12	Absent
*S. mutans*	0.24	0.48

**Table 2:** Variation in biomass reduction rate across different treatments.

	*Pfaffia paniculata* Glycolic Extract			Nystatin	Chlorhexidine
Microorganisms	0.48 mg/mL	0.96 mg/mL	1.93 mg/mL	100,000 units/mL	0.12%
*C. albicans*	40.50	41.50	45.60	34.39	
*C. dubliniensis*	3.42	11.30	23.80	16.02	
*C. glabrata*	19.80	25.25	26.06	21.80	
*C. guilhermondii*	26.70	34.70	40.00	36.37	
*C. krusei*	35.90	33.20	37.53	27.28	
*C. tropicalis*	18.64	27.47	34.27	27.08	
*P. aeruginosa*	8.30	17.69	23.53		22.61
*S. mutans*	14.30	15.63	14.72		18.18

**Table 3:** Variation in percentage of metabolic activity reduction across different treatments.

	*Pfaffia paniculata* Glycolic Extract			Nystatin	Chlorhexidine
Microorganisms	0.48 mg/mL	0.96 mg/mL	1.93 mg/mL	100,000 units/mL	0.12%
*C. albicans*	65.90	74.30	77.40	63.34	
*C. dubliniensis*	6.90	6.60	14.50	5.69	
*C. glabrata*	66.40	70.40	70.10	63.75	
*C. guilhermondii*	18.60	30.20	55.90	40.63	
*C. krusei*	29.50	12.30	16.90	29.18	
*C. tropicalis*	36.80	56.00	61.60	36.41	
*P. aeruginosa*	0.11	33.80	78.60		51.58
*S. mutans*	23.2	15.4	20.30		20.95

## Data Availability

The original contributions presented in the study are included in the article, further inquiries can be directed to the corresponding authors.
